# Validation of a portable marker-based motion analysis system

**DOI:** 10.1186/s13018-021-02576-2

**Published:** 2021-07-03

**Authors:** Shaobai Wang, Xiaolong Zeng, Liang Huangfu, Zhenyan Xie, Limin Ma, Wenhan Huang, Yu Zhang

**Affiliations:** 1 Department of Kinesiology, Shanghai University of Sport, Shanghai, China; 2 Department of Orthopaedics, Guangdong Provincial People’s Hospital, Guangdong Academy of Medical Sciences, Guangzhou, 510000 Guangdong China; 3 School of Medicine, South China University of Technology, Guangzhou, 510006 China; 4 Shantou University Medical College, Shantou, 515041 China

**Keywords:** Marker-based motion analysis, Biplanar fluoroscopy, Kinematics, Knee

## Abstract

**Background:**

The Opti_Knee system, a marker-based motion capture system, tracks and analyzes the 6 degrees of freedom (6DOF) motion of the knee joint. However, the validation of the accuracy of this gait system had not been previously reported. The objective of this study was to validate and the system. Two healthy subjects were recruited for the study.

**Methods:**

The 6DOF kinematics of the knee during flexion–extension and level walking cycles of the knee were recorded by Opti_Knee and compared to those from a biplanar fluoroscopy system. The root mean square error (RMSE) of knee kinematics in flexion–extension cycles were compared between the two systems to validate the accuracy at which they detect basic knee motions. The RMSE of kinematics at key events of gait cycles (level walking) were compared to validate the accuracy at which the systems detect functional knee motion. Pearson correlation tests were conducted to assess similarities in knee kinematic trends between the two systems.

**Results:**

In flexion–extension cycles, the average translational accuracy (RMSE) was between 2.7 and 3.7 mm and the average rotational accuracy was between 1.7 and 3.8°. The Pearson correlation of coefficients for flexion–extension cycles was between 0.858 and 0.994 for translation and 0.995-0.999 for angles. In gait cycles, the RMSEs of angular knee kinematics were 2.3° for adduction/abduction, 3.2° for internal/external rotation, and 1.4° for flexion/extension. The RMSEs of translational kinematics were 4.2 mm for anterior/posterior translation, 3.3 mm for distal/proximal translation, and 3.2 mm for medial/lateral translation. The Pearson correlation of coefficients values was between 0.964 and 0.999 for angular kinematics and 0.883 and 0.938 for translational kinematics.

**Conclusion:**

The Opti_Knee gait system exhibited acceptable accuracy and strong correlation strength compared to biplanar fluoroscopy. The Opti _Knee may serve as a promising portable clinical system for dynamic functional assessments of the knee.

**Supplementary Information:**

The online version contains supplementary material available at 10.1186/s13018-021-02576-2.

## Introduction

The motion analysis of human joints has been frequently investigated in clinical practice, such as in the areas of orthopedics, sports medicine, and rehabilitation [1]. Quantified joint kinematics data has greatly helped people to understand motion characteristics of pathology and after treatment, such as anterior cruciate ligament (ACL) deficiency and ACL reconstruction [2]. Distinct motion characteristics may serve as the scientific basis for assisted diagnosis and guidance in rehabilitation. Current motion analysis systems have several requirements, such as large space, high hardware expenses, well-trained operators, and long experiment time, which limited their clinical application [3–5].

Recently, a portable motion analysis system, Opti_Knee (Innomotion Inc, Shanghai), was developed to track and analyze the 6DOF motion of the knee joint in a convenient and user-friendly clinical setup. The kinematic characteristics of knee diseases have been widely explored using this gait analysis system, such as ACL deficiency, knee osteoarthritis, and general joint hypermobility syndrome [6–10]. However, the accuracy of this gait system has not been previously validated. With the wide application of this system, increasing attention has been paid to this issue. Hence, the objective of this study is to validate the accuracy of the Opti_Knee system.

Biplanar fluoroscopy techniques were previously reported and confirmed to have submillimeter accuracy. Giphart et al. found that biplane fluoroscopy had an average bias and precision of 0.01±0.65° for rotation and 0.01±0.59 mm for joint translation [11]. Currently, the biplane fluoroscopy technique is considered to be the gold standard for the detection of the motions of the knee joint. This technique has been used to validate the accuracy of other techniques in the determination of knee motions [12–14]. Hence, a biplanar fluoroscopy system [15] will be used to evaluate the accuracy of knee kinematic measurements from the Opti_knee gait system.

## Material and methods

### System specification and experiment setup

**Fig. 1:**
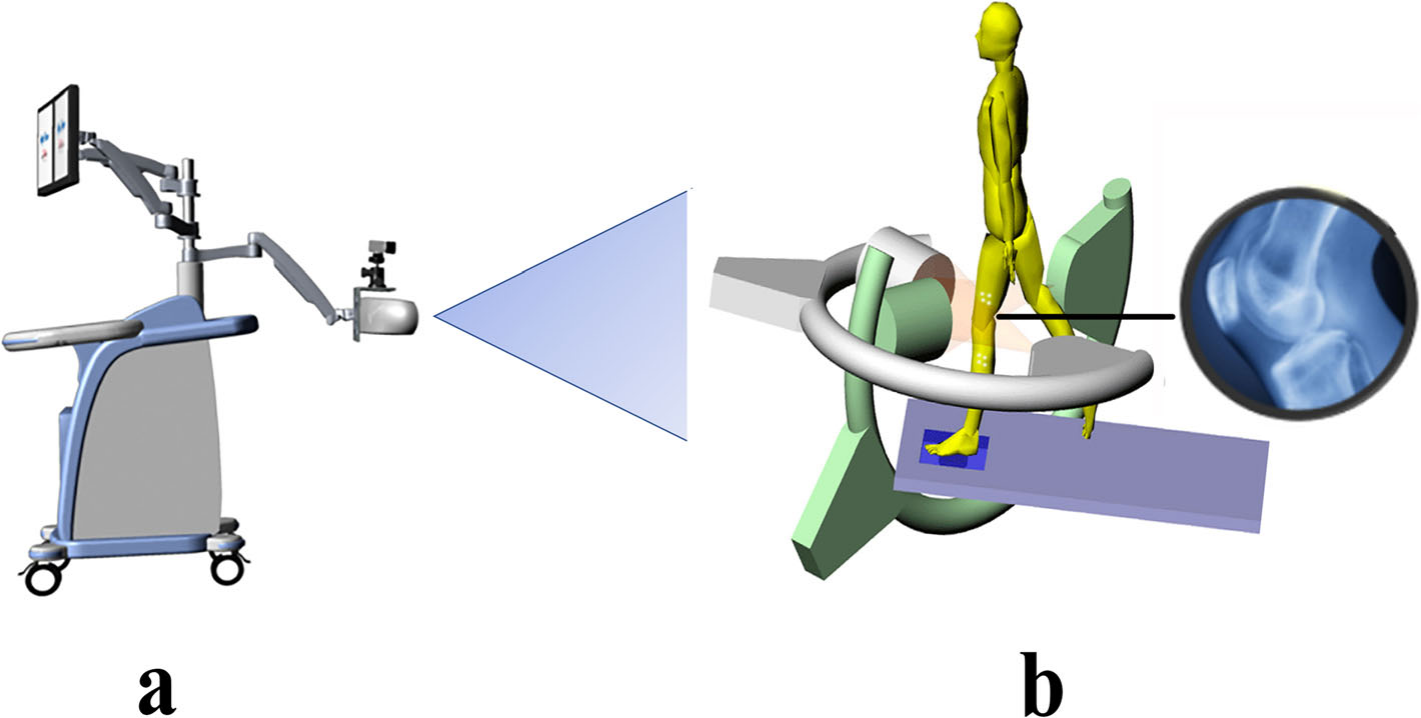
Experimental setup for gait analysis.

A biplanar fluoroscopy system (Innomotion, Inc., Shanghai) was used to calculate 6DOF knee kinematics to validate the accuracy of the Opti_Knee system (Fig. 1b). The Opti_knee gait system was used to collect gait data, and the biplanar fluoroscopy system was also used to collect the data at the same time. The biplanar fluoroscopy system captured perspective bone images from two views and calculated joint kinematics through a semi-automatic 3D–2D matching process described in great detail in several publications [15]. The biplanar fluoroscopy system is regarded as the most accurate non-invasive system because it does not require the use of skin artifacts or direct assessments of bone movement and has accuracy levels of 0.3 mm for translation and 0.6° for rotation.

**Fig. 2:**
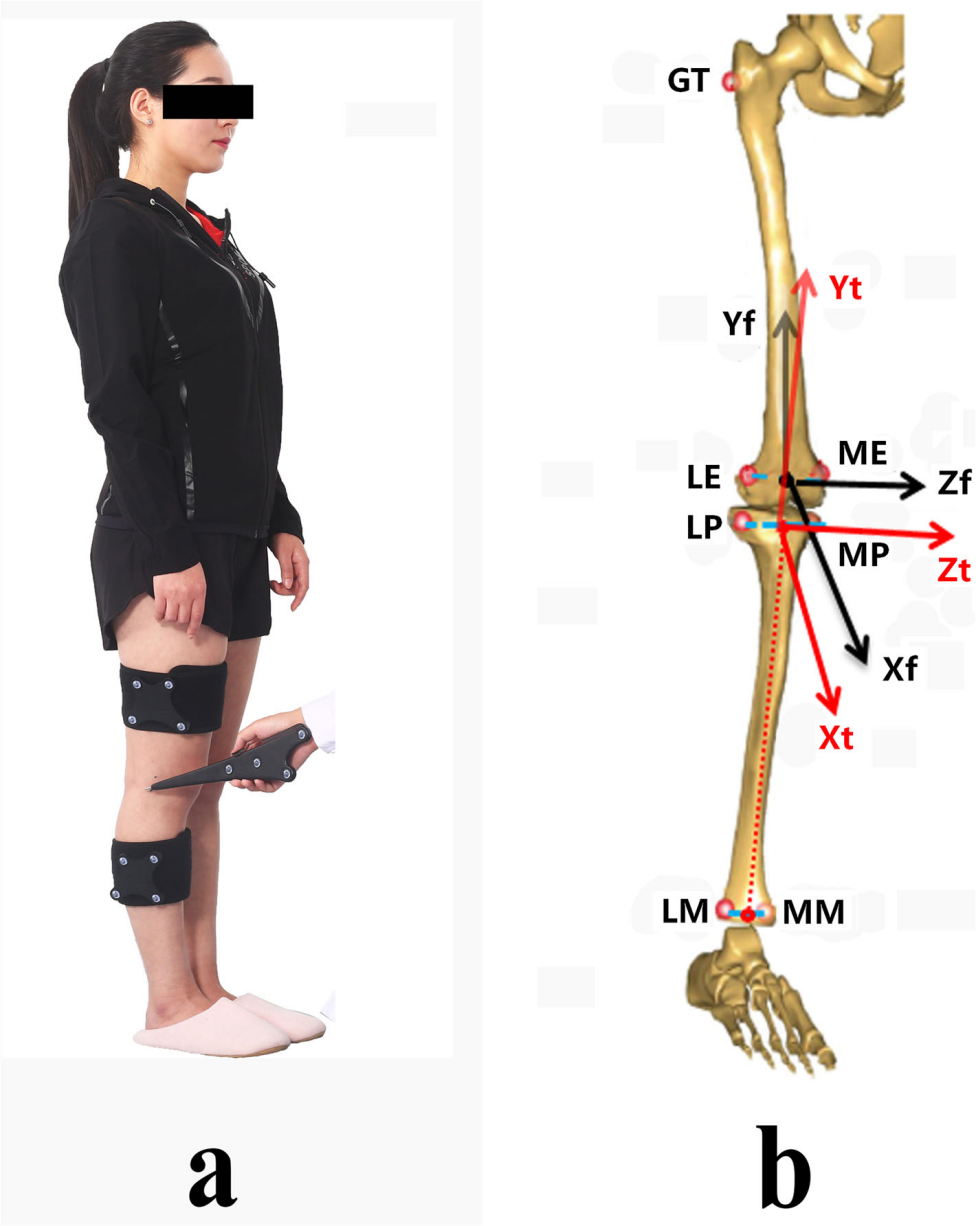
Scene of the setup of tibiofemoral coordinate systems. **a** This graph showing that the two marker sets were fixed to the thigh and shin. A digitizer was used to calibrate patient-specific bone landmark points (i.e., great trochanter (GT), medial epicondyle (ME), lateral epicondyle (LE), medial tibia plateau (MP), lateral tibia plateau (LP), medial malleolus (MM), and lateral malleolus (LM)) with the participants in a neutral standing position. **b** This graph exhibited the tibiofemeral coordinate systems. By the bony landmarkers, a tibiofemoral coordinate system was estabilshed [6]

To evaluate the accuracy of the Opti_Knee gait system when detecting knee kinematics, both the Opti_knee gait system and biplanar fluoroscopy system were used to simultaneously capture the knee motions of the subjects. The Opti_Knee system (Fig. 1a) is based on surgical navigation technology. Two high-speed inferred cameras (Polaris Spectra, Northern Digital Inc., Waterloo, Ontario, Canada) were integrated into a housing to fix the relative 3D spatial positions of the cameras. The system captures the 3D positions of points in 60 Hz, with a field of view of approximately 2 × 2 m at a distance between 2 and 3 m. The total space requirement of the system and testing area is about 10 m^2^. Two healthy subjects (2 males, age = 35 ± 0.7-year-old, height = 170.5 ± 2.1 cm, weight = 70.5 ± 4.9 kg, body mass index = 24.2±1.1 kg/m^2^) were enrolled in this study. Two marker sets were fixed to the thigh and shin (Fig. 2a). A digitizer was used to calibrate patient-specific bone landmark points (i.e., great trochanter (GT), medial epicondyle (ME), lateral epicondyle (LE), medial tibia plateau (MP), lateral tibia plateau (LP), medial malleolus (MM), and lateral malleolus (LM)) with the participants in a neutral standing position (Fig. 2b). The neutral standing position was also used as a zero reference. Data about the positions of the marker sets was collected while the knee moved. The bony landmarks were calculated by the geometric relationship setup obtained from the initial position. 6DOF knee joint kinematics were calculated based on the local coordinate systems of the femur and the tibia using the bony landmarks (Fig. 2b). The averages and standard deviations of the knee kinematics from all gait cycles were calculated using an automated program.

### Experimental procedures

The most frequent and simple movement of the knee is flexion–extension. To evaluate the accuracy of the gait system’s measurements of the basic rotational and translational movements of the knee, knee kinematics during extension–flexion cycles were determined. The functional and frequent movement was level walking. Furthermore, level walking was a widely studied motion task [7, 16–18]. Hence, the accuracy of the Opti_knee gait system when detecting the functional movements of the knee in level walking was validated using the biplane fluoroscopy system.

**Fig. 3:**
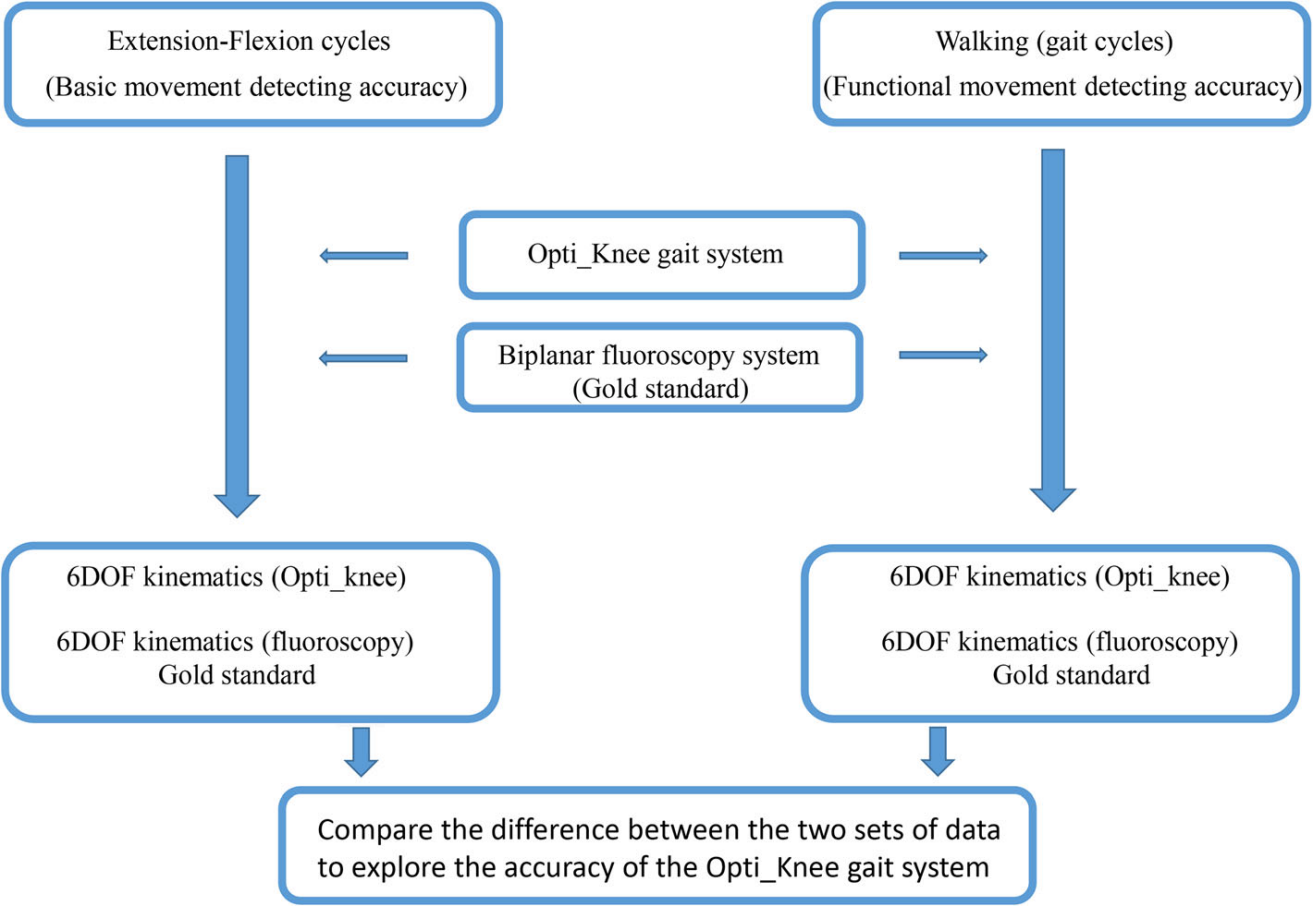
The procedure of the experiment

Two motions (simple flexion–extension and level walking) were simultaneously recorded by both the Opti_Knee and biplanar fluoroscopy systems (Fig. 3). The 6DOF knee kinematics measurements from the two systems were compared. For simple flexion–extension cycles, while sitting on a chair (1.2 m high), the subjects extended and flexed their knees from 0 to 90° for 30 s at a self-selected speed. The mean rotation accuracy and translation accuracy of flexion–extension cycle measurements were explored to determine the accuracy level of the Opti_Knee detecting gait system when measuring knee motions. For gait cycles (level walking), the subject walked on a treadmill for about 30 s at a self-selected speed. To examine the accuracy of the functional movement measurements from the Opti_knee gait system, the knee kinematics of key events were compared between the two motion capture systems, including initial contact (IC, at 1% of gait cycles), load response (LR, at 12% of gait cycles), mid-stance phase (MS, at 31% of gait cycles), toe-off (TO, 62% of gait cycles), and maximum flexion in swing phase (at 76% of swing phase) [19]. The overall setup, training (about 5 min), and experimental time (about 5 min) took around 10 min per person.

### Statistical analysis

The kinematic differences between the Opti_knee gait system and biplanar fluoroscopy system were quantified based on root mean square error (RMSE) [20] using Microsoft Office Excel (2013 version, Microsoft, Redmond, WA, USA). Bivariate Pearson correlations were calculated to compare the similarity trends between the two techniques using SPSS version 22.0 (IBM Corp., Armonk, NY, USA). The interpretation of correlation coefficients was done based on the following categories: weak (< 0.65), moderate (0.65–0.75), good (0.75–0.85), very good (0.85–0.95), and excellent (> 0.95) [21, 22].

## Results

### Validation of kinematic measurement accuracy in flexion–extension cycles

The accuracy of the Opti_knee gait system for the adduction/abduction of gait cycles was found to be 1.9°. The system’s accuracy for the internal/external rotation of gait cycles was found to be 3.8°. The accuracy of its flexion/extension measurements was found to be 1.7°. The accuracy of the Opti_knee gait system was found to be 3.3 mm for anterior/posterior translation, 3.7 mm for distal/proximal translation, and 2.7 mm for medial/lateral translation. The Pearson correlations of the coefficients of the angular DOFs were between 0.995 and 0.999, showing excellent correlations between the two motion capture systems. The Pearson correlations of the coefficients of translational DOFs were between 0.858 and 0.994, showing very good correlations between the two motion capture systems. In general, the Opti_knee gait system achieved high angular and translational accuracy and excellent correlations in detecting basic movements of the knee (flexion–extension) compared to the biplanar fluoroscopy system. The details are shown in Table 1.

**Table 1:** Accuracy of the knee kinematics determined by the Opti_Knee gait system and biplanar fluoroscopy system in flexion–extension cycles

Variables	RMSE	Mean differences	SD of differences	95% limits of agreement	R	R square
Add/Abd (°)	1.9	− 0.1	2.2	(− 4.4, 4.2)	0.995	0.990
Int/Ext rotation (°)	3.8	− 3.8	2.0	(− 7.7,0.1)	0.998	0.996
Flexion/extension (°)	1.7	− 0.9	1.7	(− 4.2, 2.4)	0.999	0.998
Ant/Pos translation (mm)	3.3	0.9	3.7	(− 6.2, 8.4)	0.858	0.736
Dis/Pro translation (mm)	3.7	0.8	3.9	(− 7.6, 8.4)	0.994	0.988
Med/Lat translation (mm)	2.7	0	3.1	(− 6.1, 6.1)	0.911	0.830

### Validation of kinematic accuracy in gait cycles

The accuracy of the Opti_knee gait system for the adduction/abduction of gait cycles was found to be 2.3°. The system’s accuracy for the internal/external rotation of gait cycles was found to be 3.2°. The accuracy of its flexion/extension measurements was found to be 1.4°. The accuracy of the Opti_knee gait system was found to be 4.2 mm for anterior/posterior translation, 3.3 mm for distal/proximal translation, and 3.2 mm for medial/lateral translation. The Pearson correlations of the coefficients of the angular DOFs were between 0.964 and 0.999, showing excellent correlations between the two motion capture systems. The Pearson correlations of the coefficients of translational DOFs were between 0.883 and 0.938, showing very good correlations between the two motion capture systems. The Opti_knee gait system achieved high accuracy and very good in translational DOFs in gait cycles compared to the biplanar fluoroscopy system. The angular accuracy and correlations values were higher than those for translational accuracy and correlations in gait cycles.

## Discussion

With the gradual popularization of the Opti_knee gait system, we will inevitably need to clarify the accuracy of this gait system. The Opti_knee gait system exhibited high accuracy for both angular (RMSE: 1.7–3.8°) and translational (RMSE: 2.7–3.7 mm) DOFs and “very good” to “excellent” correlations (0.858–0.999) compared to biplanar fluoroscopy system in flexion–extension cycles (Table 1). This finding suggests that the Opti_knee gait system has a high degree of accuracy in detecting the basic movements of the knee. Level walking is the most frequent and functional motion of the knee. Further, our finding showed that the Opti_knee gait system has high accuracy (RMSE: 1.4–3.2° for angular DOFs, 3.2–4.2 mm for translational DOFs) and strong positive correlation (0.964–0.999, excellent for angular DOFs; 0.883–0.938, very good for translational DOFs) in level walking (gait cycles) compared to the biplanar fluoroscopy system. These findings suggest that the accuracy of the system is acceptable compared to other marker-based motion analysis systems [13, 23–25].

Marker-based motion capture systems are presently the most frequent methods used to determine tibiofemoral kinematics. Considering the RMSE of the marker-based motion capture system, skin artifact movement is always an inherent problem, especially in the translational movement. Compared to fluoroscopy techniques, it was reported that the translation of skin artifact was between 10 and 30 mm of relative motion in marker-based motion capture system [14]. However, our results showed that the Opti_knee gait system had high accuracy (RMSE: 1.4–3.2° for angular DOFs, 3.2–4.2 mm for translational DOFs). The accuracy of the Opti_knee gait system was higher it was previously reported to be [13, 23–25]. This could be related to improvements to the motion capture techniques. The tracking errors of the gait system can be mainly divided into two parts, the ability of the tracking systems to detect movement and the artifact of soft tissue. The accuracy of the tracking system (NDI Polaris Spectra) used in the Opti_knee gait system [7] was 0.3 mm [26]. Hence, improvements to the tracking ability could explain the increased accuracy of the Opti_knee gait system when determining knee kinematics.

The findings suggest “very good” (0.883–0.938 for translational DOFs) and “excellent” (0.964–0.999 for angular DOFs) correlations between the Opti_knee gait system and the biplanar fluoroscopy system (Table 2). This is meaningful and of great importance in the clinical application of gait systems. In fact, avoiding talking about measurement errors between the Opti_knee gait system and biplanar fluoroscopy system, we can compare the two techniques to two clinical scales, like the International Knee Documentation Committee (IKDC) and Lysholm scales for knee injury [27] or American Knee Society Score (AKS) and Western Ontario and McMaster Universities Osteoarthritis Index (WOMAC) for knee osteoarthritis [28]. Although the content of these scales differs, their core aims are to assess the functional abilities of patients. If one of the scales was considered to be the gold standard in the assessment of certain diseases and the other scale was effective and highly correlated to the “gold standard” scale, then clinically, both scales could be treated as having the same effectiveness in the assessment of diseases [29, 30]. Hence, to an extent, the findings of this study suggest that the Opti_knee gait system has similar abilities as the biplanar fluoroscopy system in the assessment of knee kinematics. For example, patients with ACL deficiency were found to have increased tibial translation using the biplanar fluoroscopy system [31, 32]. Marker-based motion capture systems could also be used to identify increased anterior tibial translation in patients with ACL deficiency [7, 33].

**Table 2:** Accuracy of knee kinematics determined by the Opti_Knee gait system and biplanar fluoroscopy system in gait cycles

Variables	RMSE	Mean differences	SD of differences	95% limits of agreement	R	R square
Add/Abd (°)	2.3	− 0.7	2.2	(− 5.2, 3.8)	0.979	0.958
Int/Ext rotation (°)	3.2	0.6	3.1	(− 5.5, 6.7)	0.964	0.929
Flexion/extension (°)	1.4	− 0.1	1.4	(− 2.8, 2.6)	0.999	0.998
Ant/Pos translation (mm)	4.2	− 0.1	4.2	(− 8.3, 8.1)	0.938	0.880
Dis/Pro translation (mm)	3.3	− 1.6	2.8	(− 7.1, 3.9)	0.883	0.780
Med/Lat translation (mm)	3.2	− 2.3	2.3	(− 6.8, 2.2)	0.912	0.832

Notes: *RMSE* root mean square error, *SD* standard deviation, *Add/Abd* abduction/adduction, *Int/Ext* internal/external, *Flex/Ext* flexion/extension, *Ant/Pos* anterior/posterior, *Dis/Pos* distal/proximal, *Med/Lat* medial/lateral

The portable Opti_Knee system has the advantages of small space requirements, user-friendliness, and short testing time. With surgical navigation hardware, the accuracy of the system is comparable to those of conventional motion analysis systems. The average translational accuracy (RMSE) was found to be 3.6 mm, and rotational accuracy was found to be 2.3° in level walking. The study also showed very good to excellent correlation (0.883–0.999) of dynamic knee function assessment between Opti_Knee gait system and biplanar fluoroscopy system. There could be some limitations in the Opti_Knee gait system. Firstly, one limitation of the system is its relatively small field of view, which is still enough to assess a single joint’s kinematics in a well-planned clinical setup. Secondly, although the accuracy of the Opti_Knee gait system was found to be high enough for the clinical assessment of knee kinematics to identify knee diseases, due to inherent soft tissue artifacts, it can only provide rough (i.e., not exact guidance) for some “intact” situations, especially surgical procedures and intact motion-reappearance-based biomechanical experiments. These situations require at least submillimeter accuracy to cure patients or restore intact motion. Bone-pin markers (invasive) must be used to get rid of soft tissue artifacts [24, 34]. The Opti _Knee gait system may serve as a promising portable clinical system for dynamic and functional assessments of the knee.

## Conclusion

A high degree of accuracy for rotation and translation measurement and very good (translation) to excellent (angle) correlation strengths were achieved in the Opti_Knee gait system compared to the biplanar fluoroscopy systems. Therefore, the Opti_knee gait system can provide acceptable accuracy for the clinical determination of knee kinematics.

## Supplementary Information

**Additional file 1.**

## Data Availability

The datasets used or analyzed during the current study are available from the corresponding author on reasonable request.

## Abbreviations

RMSE: Root mean square error
6DOF: Six degrees of freedom
ACL: Anterior cruciate ligament
GT: Great trochanter
ME: Medial epicondyle
LE: Lateral epicondyle
MP: Medial tibia plateau
LP: Lateral tibia plateau
MM: Medial malleolus
LM: Lateral malleolus
IKDC: The International Knee Documentation Committee
AKS: American Knee Society Score
WOMAC: Western Ontario and McMaster Universities Osteoarthritis Index
IC: Initial contact
LR: Load response
MS: Mid-stance phase
TO: Toe-off
