# Supplementary material for: Validation of a portable marker-based motion analysis system
Source: J Orthop Surg Res. 2021 Jul 3;16:425. doi: 10.1186/s13018-021-02576-2 (PMC8254326; doi:10.1186/s13018-021-02576-2)
Supplement: Supplementary file 1 — Additional file 1. [file 13018_2021_2576_MOESM1_ESM.docx]

To test the reliability of measurement of the gait system, a test-retest experiment was performed during gait cycle. Ten subjects (five males and five females, 23.6±2.5 years old, 168.0±7.1cm, 56.4±8.9 kg) were enrolled in the experiment. Subjects were measured by the gait system for two days. The intraclass correlation coefficient (ICC) was performed by the MedCalc Statistical Software version 15.8 (MedCalc Software bvba, Ostend, Belgium). An ICC value of >0.75 represents good reliability[1]. The standard error of measurement (SEM) was calculated with the equation SD*SQRT (1-ICC) using Excel 2010 (Microsoft, Redmond, WA)[2].

The reliability indices of the parameters were given in Table 1&2. The ICCs of the rotational parameters were between 0.9225 and 0.9933 (Table 1). The ICCs of the translational parameters were between 0.9673 and 0.9950 (Table 2). Both sets of results indicated good test-retest reliability of knee kinematics. The overall SEM was low for the kinematics (mean ± standard deviation, 0.7±0.4 degree for rotation and 0.5±0.1 mm for translation). In angular parameters, the SEM ranged between 0.2 and 1.7 degree (Table 1). In translational parameters, the SEM ranged between 0.4 and 0.7 mm (Table 2). All above, this experiment showed good reliability of kinematic measurement by Opti_Knee gait system.

Table 1 the reliability of rotation parameters of the gait system.

| Parameters (Rotation) | | Day 1 | Day 2 | ICC (95% CI) | SEM |
| --- | --- | --- | --- | --- | --- |
|  |  | Mean(SD) | Mean(SD) |  |  |
| Add/Abd (°) | IC | -0.2(1.8) | -0.2(1.8) | 0.9732(0.9322-0.9894) | 0.3 |
|  | LR | -1.5(1.7) | -1.4(1.8) | 0.9784(0.9455-0.9915) | 0.2 |
|  | MS | -2.2(1.6) | -2.4(1.8) | 0.9913(0.9781-0.9966) | 0.2 |
|  | TO | -2.6(2.9) | -2.4(2.7) | 0.9819(0.9543-0.9928) | 0.4 |
|  | MFSP | 1.4(3.6) | 1.1(3.6) | 0.9928(0.9819-0.9972) | 0.3 |
| Int/Ext (°) | IC | 0.6(3.8) | -0.1(3.5) | 0.9764(0.9404-0.9907) | 0.6 |
|  | LR | -0.8(3.9) | -1.1(4.0) | 0.9711(0.9270-0.9886) | 0.7 |
|  | MS | -0.2(4.2) | -0.2(4.4) | 0.9736(0.9332-0.9895) | 0.7 |
|  | TO | -2.3(5.9) | -2.8(5.8) | 0.9840(0.9849-0.9976) | 0.7 |
|  | MFSP | -6.1(7.2) | -6.7(6.9) | 0.9933(0.9831-0.9974) | 0.6 |
| Fle/Ext (°) | IC | 9.7(8.5) | 10.5(9.0) | 0.9870(0.9671-0.9948) | 1.0 |
|  | LR | 10.7(8.2) | 11.0(8.4) | 0.9878(0.9691-0.9952) | 0.9 |
|  | MS | 6.5(5.3) | 6.9(5.5) | 0.9484(0.8697-0.9796) | 1.2 |
|  | TO | 33.2(5.7) | 31.3(6.5) | 0.9225(0.8042-0.9693) | 1.7 |
|  | MFSP | 63.2(5.7) | 62.5(5.2) | 0.9611(0.9018-0.9846) | 1.1 |

Notes: Add/Abd: adduction/abduction; Int/Ext: internal/external rotation; Fle/Ext: flexion/extension; IC: initial contact; LR: load response; MS: mid-stance; TO: toe-off; NFSP: maximum flexion in swing phase; SD: standard deviation.

Table 2 the reliability of translational parameters of the gait system.

| Parameters (Translation) | | Day 1 | Day 2 | ICC (95%CI) | SEM |
| --- | --- | --- | --- | --- | --- |
|  |  | Mean (SD) | Mean (SD) |  |  |
| Ant/Pos (mm) | IC | 5.4(3.0) | 5.4(3.2) | 0.9745(0.9357-0.9899) | 0.5 |
|  | LR | 2.5(3.0) | 2.5(3.0) | 0.9688(0.9211-0.9876) | 0.5 |
|  | MS | 0.1(3.3) | 0.5(3.4) | 0.9678(0.9187-0.9873) | 0.6 |
|  | TO | -3.2(4.3) | -2.9(4.0) | 0.9831(0.9572-0.9933) | 0.5 |
|  | MFSP | 3.2(4.1) | 2.4(4.1) | 0.9845(0.9609-0.9939) | 0.5 |
| Dis/Pro (mm) | IC | 5.4(3.3) | 5.3(3.2) | 0.9654(0.9126-0.9863) | 0.6 |
|  | LR | 2.4(3.3) | 2.5(3.1) | 0.9693(0.9225-0.9879) | 0.6 |
|  | MS | 0.4(2.5) | 0.9(2.7) | 0.9707(0.9261-0.9884) | 0.4 |
|  | TO | -1.9(3.9) | -1.8(3.6) | 0.9697(0.9235-0.9880) | 0.6 |
|  | MFSP | 3.8(7.0) | 3.9(6.5) | 0.9950(0.9874-0.9980) | 0.5 |
| Med/Lat (mm) | IC | 0.7(4.3) | 1.1(4.3) | 0.9880(0.9698-0.9953) | 0.5 |
|  | LR | 0.9(3.7) | 1.4(3.8) | 0.9799(0.9493-0.9921) | 0.5 |
|  | MS | 0.2 (3.7) | 0.2(3.7) | 0.9756(0.9385-0.9904) | 0.6 |
|  | TO | 2.3(3.6) | 2.0(3.6) | 0.9673(0.9173-0.9870) | 0.6 |
|  | MFSP | -0.8 (4.6) | -0.8(5.1) | 0.9806(0.9510-0.9923) | 0.7 |

Notes: Ant/Pos: anterior/posterior; Dis/Pro: Distal/Proximal; Med/Lat: medial/lateral; IC: initial contact; LR: load response; MS: mid-stance; TO: toe-off; NFSP: maximum flexion in swing phase; SD: standard deviation.

**References**

1. Kramer, M.S. and A.R. Feinstein. Clinical biostatistics. LIV. The biostatistics of concordance. Clin Pharmacol Ther. 1981; 29(1): 111-23.

2. Atkinson, G. and A.M. Nevill. Statistical methods for assessing measurement error (reliability) in variables relevant to sports medicine. Sports Med. 1998; 26(4): 217-38.
